# Total bilirubin and bilirubin-to-triglycerides ratio predict changes in glycated hemoglobin in healthy children

**DOI:** 10.3389/fendo.2023.1303597

**Published:** 2023-12-01

**Authors:** Elsa Puerto-Carranza, Silvia Nuevo-Casals, Berta Roca-Portella, Berta Mas-Parés, Ariadna Gómez-Vilarrubla, Gemma Carreras-Badosa, Maria Niubó, Anna Prats-Puig, Francis de Zegher, Lourdes Ibáñez, Judit Bassols, Abel López-Bermejo

**Affiliations:** ^1^Pediatric Endocrinology Group, Girona Biomedical Research Institute (IDIBGI), Salt, Spain; ^2^Pediatrics, Dr. Trueta University Hospital, Girona, Spain; ^3^Maternal-Fetal Metabolic Group, Girona Biomedical Research Institute (IDIBGI), Salt, Spain; ^4^University School of Health and Sport (EUSES), University of Girona, Girona, Spain; ^5^Department of Development & Regeneration, University of Leuven, Leuven, Belgium; ^6^Endocrinology, Pediatric Research Institute, Sant Joan de Déu Children’s Hospital, Esplugues, Barcelona, Spain; ^7^Spanish Biomedical Research Centre in Diabetes and Associated Metabolic Disorders (CIBERDEM), ISCIII, Madrid, Spain

**Keywords:** bilirubin, triglycerides, glycated hemoglobin, children, pediatrics

## Abstract

**Objective:**

Bilirubin and triglycerides can regulate insulin secretion and glucose uptake. The aim of our study is to analyze associations between total bilirubin (TB) and the bilirubin-to-triglycerides ratio (BTR) with metabolic markers in healthy prepubertal children.

**Methods:**

Subjects were 246 healthy children (mean age 8), of whom 142 (58%) were reevaluated 4 years later (mean age 12). The subjects were stratified according to age into three groups (<7.8 years; 7.8-9.6 years; and >9.6 years; n=82 each) at baseline and into two groups (<12.9 years and ≥12.9 years; n=71 each) at follow-up. Anthropometrics and laboratory parameters [TB and its fractions (direct and indirect bilirubin), triglycerides, HDL-cholesterol, glucose, insulin, HOMA-IR, HOMA-B and glycated hemoglobin (HbA1c)] were assessed at both baseline and follow-up.

**Results:**

TB and BTR showed independent and negative association with baseline and follow-up HbA1c. These associations were stronger for BTR and in the highest age group. No independent associations were observed with HOMA-IR or HOMA-B.

**Conclusion:**

TB and BTR are independently associated with HbA1c and predict its changes over time in healthy children. Our results indicate that TB and BTR may be useful parameters in studies of glucose tolerance in healthy children.

## Introduction

Bilirubin is an abundant molecule present in human plasma. It was firstly considered as a toxic waste product and a marker of hepatobiliary disease or the cause of kernicterus and brain damage in neonates. However, later studies showed that high-normal or moderately increased bilirubin levels may have beneficial effects thanks to its anti-oxidant, cytoprotective and anti-inflammatory properties (1, 2). Indeed, several epidemiological studies have suggested inverse associations between bilirubin and metabolic syndrome (3–8) diabetes mellitus (9–11), hypertension (12), coronary artery disease (13) and cardiovascular disease (14).

While bilirubin is a protective factor, dyslipidemia is tightly related to insulin resistance and metabolic syndrome. The effects of insulin resistance on lipoprotein metabolism have been extensively studied; however, there is increasing evidence that hypertriglyceridemia and low HDL cholesterol are possible causes of insulin resistance as well (15, 16).

In children, a number of cross-sectional studies (17–20) have shown that there is an inverse correlation between serum bilirubin, insulin resistance and metabolic syndrome. A positive relationship between triglycerides, insulin resistance and metabolic syndrome has also been reported in healthy children (21, 22) or in obese children (23, 24); however, longitudinal studies that associate total bilirubin (TB) or the bilirubin-to-triglycerides ratio (BTR, derived from the ratio between TB and triglycerides) are lacking. We hypothesize that TB and BTR will be associated with metabolic markers in apparently healthy children and assess this hypothesis in both cross-sectional and longitudinal studies.

## Materials and methods

### Subjects and ethics

Subjects were 246 healthy children (116 girls and 130 boys, age 8.8 ± 0.1) from a cohort study designed to assess cardiovascular risk factors in children in Northern Spain, of whom 142 (58%) were reevaluated 4 years later (68 girls and 74 boys, age 12.9 ± 0.1). No differences were observed for baseline characteristics between children with and without follow-up. For the purpose of the present study, at baseline the children were categorized into three groups according to tertiles of age: <7.8 years; between 7.8 and 9.6 years; and >9.6 years (n=82 for each group). Because of the smaller sample size, at follow-up the children were categorized into two age groups, according to the median value: < 12.9 years and ≥12.9 years (n=71 for each group).

Inclusion criteria were: 1) Caucasian origin; 2) age between 5 and 12 years at baseline. Exclusion criteria were: 1) major congenital anomalies; 2) evidence of chronic illness or prolonged use of any kind of medication; 3) acute illness or use of medications in the month preceding potential enrollment; 4) bilirubin higher than 1.2 mg/dl; 5) HbA1c higher than 6.5% (48 mmol/mol); 6) triglycerides higher than 200 mg/dl; 7) systolic blood pressure and/or diastolic blood pressure higher than 97^th^ percentile for age, gender and height; 8) HDL cholesterol lower than 35 mg/dl.

The study protocol was approved by the Institutional Review Board of Dr. Josep Trueta Hospital. Informed written consent was obtained from parents.

### Clinical and laboratory data

Clinical and laboratory examinations were performed between 8:00 and 9:00 AM. Height was measured with a Harpenden stadiometer (Holtain) and weight was measured wearing light clothes with a calibrated scale (Seca). Body mass index (BMI) was calculated using the formula: weight (kg)/height (meters)^2^. Weight, height and BMI were transformed to Z-score values using regional data (25). Waist circumference was measured at the umbilical level. Puberty was assessed by a trained nurse using Marshall and Tanner criteria (26, 27). Blood pressure (BP) was measured in the supine position on the right arm after 10 minutes rest using an electronic sphygmomanometer (Dinamap Pro 100, GE Healthcare) with cuff size appropriate for arm circumference and the average of three readings were recorded in each subject.

All serum samples were obtained under fasting conditions. Serum glucose was analyzed by the hexokinase method. Insulin was measured by immunochemiluminiscence (Immulite2000, Siemens); the detection limit was 0.4 mIU/l and the inter- and intra-assay coefficient of variation (CVs) were lower than 10%. Insulin resistance was estimated from fasting insulin and glucose levels using the homeostasis model assessment index (HOMA-IR) calculated with the formula: fasting insulin (µIU/ml) x fasting glucose (mg/dl)/405. Homeostasis model assessment of beta-cell function (HOMA-B) was calculated with the formula: 20 × fasting insulin (µU/ml)/[fasting plasma glucose (mg/dl) – 63]. HbA1c was measured using the standardized method Diabetes Control and Complications Trial (DCCT)/International Federation of Clinical Chemistry (IFCC) (Menarini). Total cholesterol was measured using colorimetric enzymatic method (Cobas 702, Roche); HDL cholesterol was quantified by homogenous method of selective detergent with accelerator (ARCHITECHT, Abbot Laboratories); and LDL was calculated with Fridewald formula. Total serum triglycerides were measured by monitoring the reaction of glycerol-phosphate-oxidase method (ARCHITECTH, Abbot Laboratories); the detection limit was 5 mg/dl and CVs lower than 5%. The triglyceride-to-HDL ratio was calculated as TG divided by HDL. TB and DB were measured using the 2-4 and 2-5 diazoateddichloroaniline method (Roche); the detection limit was 0.1 mg/dl and the CVs lower than 10% for both. IB was obtained by subtracting absolute DB values from absolute TB values. The BTR (%) was calculated as TB value divided by triglycerides value and multiplied by 100.

### Statistics

Statistical analyses were performed using SPSS version 22.0 (SPSS Inc, Chicago, IL, USA). Results for Gaussian variables are expressed as mean and SEM and for non-Gaussian variables as median and interquartile range. Non-Gaussian variables were mathematically transformed to logarithm to improve symmetry. Differences in continuous variables among age groups were examined by ANOVA. The relation between variables was analyzed by Pearson correlation followed by multiple regression analysis (the stepwise method being used for computing effect sizes of the observed associations). Significance level was set at p< 0.05. The interaction of age in the association between HbA1c and both TB and BTR was tested using ANCOVA analysis. A relevant interaction was considered with p values < 0.15 (28). Finally, logistic binary regression analyses were used to estimate the odds ratio (OR) and 95% confidence interval (CI) of having a follow-up HbA1c value above 5.3 (the median calculated for the studied subjects) based on baseline TB and BTR values. Receiver operating characteristic (ROC) curve analyses were done to evaluate the usefulness of TB and BTR to predict follow-up HbA1c levels and distinguish between two diagnostic groups (HbA1c≥5.3 and HbA1c<5.3).

## Results

Results for clinical and laboratory assessments at baseline (n=246) are described in **Supplementary Table 1** in all the studied subjects as a whole and in subgroups thereof according to age tertiles. Follow-up results are shown in **Supplementary Table 2** for the whole sample (n=142) and for groups according to median of age. As expected, significant differences were observed among age groups for most clinical data. Total, direct and indirect bilirubin and BTR showed no differences among groups.

### Associations between bilirubin and metabolic parameters

**Figure 1:**
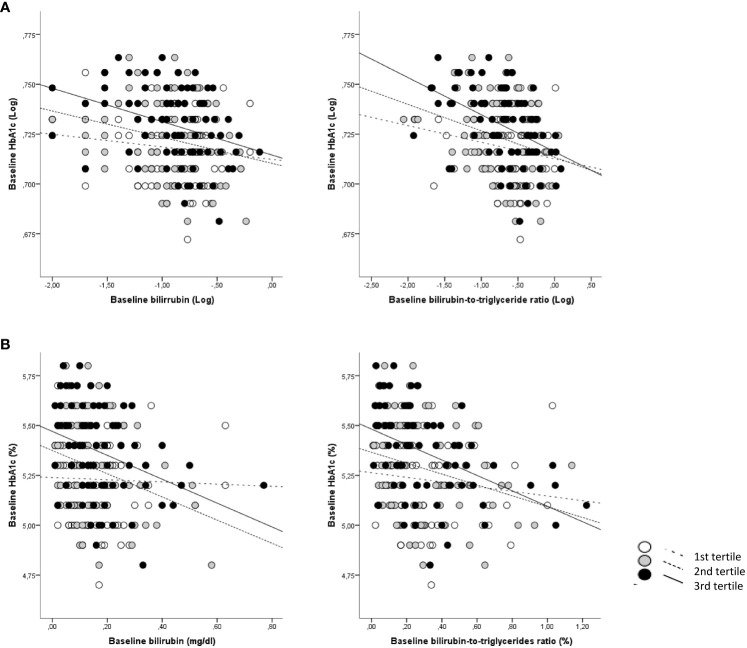
Correlations between baseline total bilirubin and BTR with baseline HbA1c according to age categories. **(A)** Log-transformed data for bilirubin and BTR are shown; **(B)** Absolute data for bilirubin and BTR are shown. White dots and dotted lines depict children <7.9 years (1st tertile); grey dots and dashed lines depict children between 7.9 and 9.6 years (2nd tertile) and black dots and solid lines depict children >9.6 years (3^rd^ tertile).

**Figure 2:**
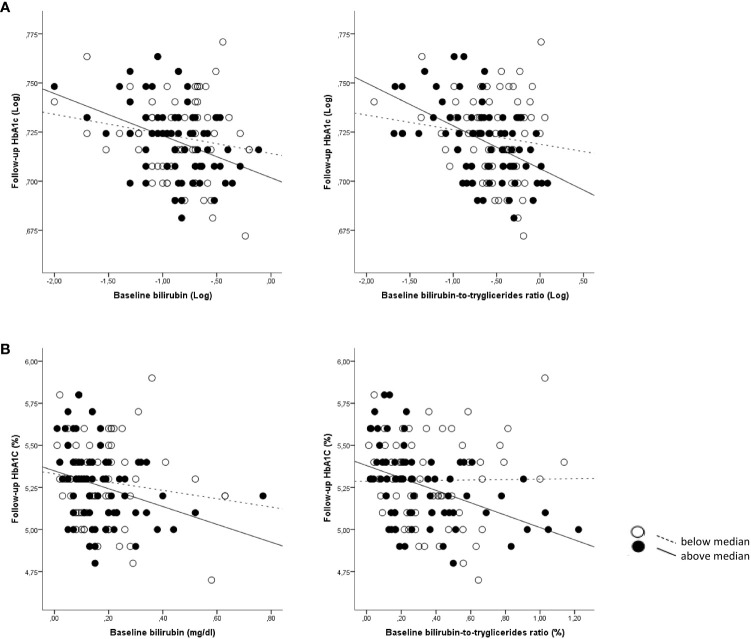
Correlations between baseline bilirubin and BTR with follow-up HbA1c according to age categories. **(A)** Log-transformed data for bilirubin and BTR are shown; **(B)** Absolute data for bilirubin and BTR are shown. White dots and dotted lines depict children <12.9 years (below median) and black dots and solid lines depict children >12.9 years (above median).

Baseline TB levels associated with a more favorable metabolic profile at baseline including lower BMI-SDS, insulin, HOMA-IR, HOMA-B, HbA1c and higher HDL-cholesterol (all p<0.05) (**Supplementary Table 3**). The association with HbA1c increased progressively across age tertile groups, showing the strongest and significant association in the third tertile (r=−0.334; p=0.002; **Supplementary Table 3** and **Figure 1**). Baseline TB levels also associated with a more favorable metabolic profile at follow-up including lower insulin, HOMA-IR, HOMA-B, HbA1c, triglycerides and TG/HDL (**Supplementary Table 3**). Again, the association with HbA1c was stronger in the highest age group (r=−0.377; p=0.001; **Supplementary Table 3** and **Figure 2**).

In multivariate linear regression analyses adjusting for confounding variables (age, gender, puberty, BMI, waist, HOMA-IR), baseline TB showed independent associations with baseline HbA1c (β= −0.220; p=0.001; R^2^ = 3.9%) (**Table 1**). This independent association proved to be stronger in the 2^nd^ age tertile (β= −0.284; p=0.019; R^2^ = 6.7%) and especially so in the 3^rd^ age tertile (β= −0.284; p=0.019; R^2^ = 10.1%). In the follow-up sample, baseline TB was an independent predictor of follow-up HbA1c (β= −0.275; p=0.002; R^2^ = 6.8%) after adjusting for the same confounding variables (**Table 1**). This association with HbA1c was stronger in the higher age group (β= −0.417; p=0.002; R^2^ = 15.8%). The effect sizes of the observed associations were as follows: per each 1 unit-increase in baseline total bilirubin (mg/dL), follow-up HbA1c decreased by 0.4% ± 0.2 (p=0.018) in the total population, and by 0.5% ± 0.2 (p=0.019) in the higher age group (**Supplementary Table 4**).

**Table 1:** Multivariate linear models of baseline and follow-up HbA1c as dependent variable and TB as independent variable in the studied subjects as a whole and in subgroups thereof according to age.

Baseline HbA1c	All subjects (n=246)			1^st^Tertile <7.8 years (n=82)			2^nd^Tertile 7.8-9.6 years (n=82)			3^rd^Tertile >9.7 years (n=82)			
Baseline parameters	β	p	R^2^	β	p	R^2^	β	p	R^2^	β		p	R^2^
TB	-0.220	0.001	**3.9**	–	–	–	-0.284	0.019	**6.7**	-0.284		0.019	**10.1**
BMI	0.167	0.032	7.8	–	–	–	–	–	–	–		–	–
Age	0.174	0.025	1.7	–	–	–	–	–	–	–		–	–
R^2^			13.4						6.7				10.1
Follow up HbA1c	All subjects (n=142)			Below median <12.9 years (n=71)				Above median ≥12.9 years (n=71)					
aseline parameters	β	p	R^2^	β		p	R^2^	β			p	R^2^	
TB	-0.275	0.002	**6.8**	–		–	–	-0.417			0.002	**15.8**	
R^2^			6.8									15.8	

Non-predictive variables at baseline: gender, puberty, waist, HOMA-IR. Non-predictive variables at follow-up: age, gender, puberty, BMI, waist, HOMA-IR. Significant results are shown in bold.

No independent associations were found between baseline bilirubin fractions and either baseline or follow-up HbA1c or with any of the bilirubin measurement and either HOMA-IR or HOMA-B at baseline or follow-up.

**Figure 3:**
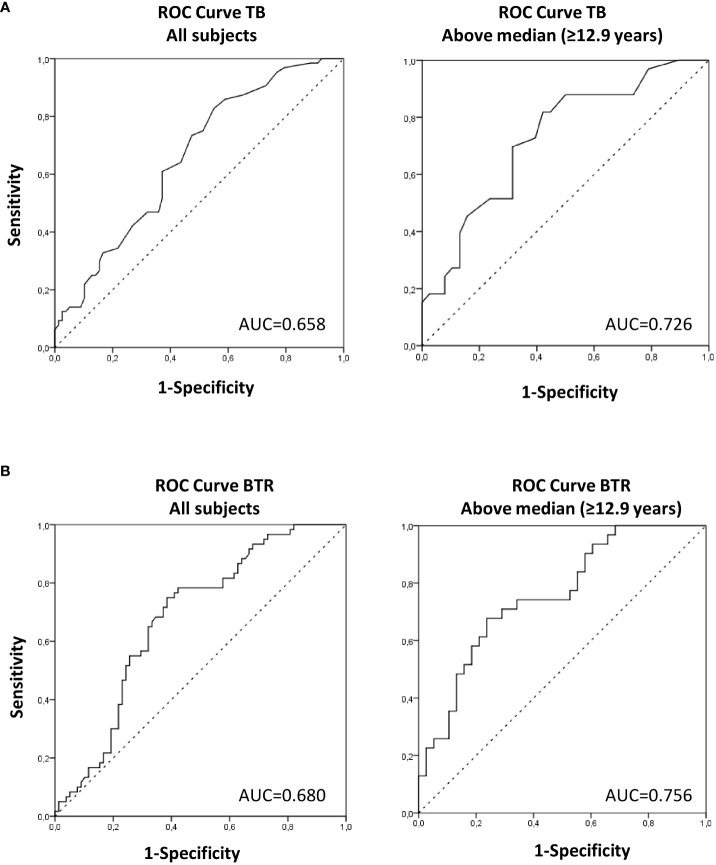
Area under the ROC curve (AUC) of TB **(A)** and BTR **(B)** to predict HbA1c at follow-up in the studied subjects as a whole and in children >12.9 years (above median). AUC between 0.59–0.74 indicate acceptable discriminatory accuracy and AUC between 0.75–0.9 indicate good discriminatory accuracy.

In a logistic binary regression analysis, the OR for having a follow-up HbA1c value > 5.3% (34 mmol/mol) (i.e., the median value for all the studied subjects) was 0.337 (95% CI 0.163-0.697; p=0.003) in subjects with baseline TB above the median for the whole group, adjusted for baseline age and BMI. In ROC curve analysis, TB was found to have an acceptable discriminatory accuracy for follow-up HbA1c prediction in all subjects (AUC of 0.658) and in the higher age group (AUC of 0.726) (**Figure 3A**).

### Associations between bilirubin-to-triglycerides ratio and metabolic parameters

The above-mentioned associations were stronger when both bilirubin and triglycerides levels were taken into account using the bilirubin-to-triglycerides ratio (BTR). Thus baseline BTR showed negative associations with baseline weight-SDS, BMI-SDS, waist, SBP, DBP, glucose, insulin, HOMA-IR, HOMA-B, HbA1c (all p<0.0001). Baseline BTR also showed negative associations with follow-up weight-SDS, BMI-SDS, waist, SBP, DBP, glucose, insulin, HOMA-IR, HOMA-B, HbA1c (all p ≤ 0.01) (**Supplementary Table 5**). Again, the association with HbA1c increased across age tertile groups, showing the strongest association in the highest age group (baseline: r= −0.452; p<0.0001; follow-up: r=-0.479; p<0.0001) (**Supplementary Table 5** and **Figures 1**, **2**).

The associations with HbA1c remained significant in multivariate regression analyses adjusting for possible confounding variables (age, gender, puberty, BMI, waist, HOMA-IR). Baseline BTR showed independent associations with baseline HbA1c in the whole population (β= −0.299; p<0.00001; R^2^ = 10.5%) and were stronger in the 2^nd^ tertile (β= −0.325; p=0.007; R^2^ = 9.2%) and especially in the 3^rd^ tertile (β= −0.428; p<0.0001; R^2^ = 17.0%) of age (**Table 2**). Moreover, baseline BTR was an independent predictor of follow-up HbA1c in the whole population (β= −0.246; p=0.004; R^2^ = 8.4%), again this being stronger in the higher age group (β= −0.486; p<0.0001; R^2^ = 22.5%) (**Table 2**). The effect sizes of the observed associations were as follows: per each 1 unit-increase in baseline BTR (%), follow-up HbA1c decreased by 0.2% ± 0.1 (p=0.014) in the total population, and by 0.4% ± 0.1 (p=<0.001) in the higher age group (**Supplementary Table 6**).

**Table 2:** Multivariate linear models of baseline and follow-up HbA1c as dependent variable and BTR as independent variable in the studied subjects as a whole and in subgroups thereof according to age.

Baseline HbA1c	All subjects (n=246)			1^st^Tertile <7.8 years (n=82)			2^nd^Tertile 7.8-9.6 years (n=82)			3^rd^Tertile >9.7 years (n=82)		
Baseline parameters	β	p	R^2^	β	p	R^2^	β	p	R^2^	β	p	R^2^
**BTR**	-0.299	<0.0001	**10.5**	–	–	–	-0.325	0.007	**9.2**	-0.428	**<0.0001**	**17.0**
BMI	–	–	–	–	–	–	–	–	–	–	–	–
Age	0.233	<0.0001	5.0	–	–	–	–	–	–	–	–	–
R^2^			15.5						9.2			17.0
Follow up HbA1c	All subjects (n=142)			Below median <12.9 years (n=71)						Above median ≥12.9 years (n=71)		
Baseline parameters	β	p	R^2^	β			p	R^2^		β	p	R^2^
**BTR**	-0.246	0.004	**8.4**	–			–	–		-0.486	<0.0001	**22.5**
BMI	0.248	0.011	3.7	–			–	–		–	–	–
Age	-0.284	0.002	2.1	–			–	–		–	–	–
R^2^			14.2									22.5

Non-predictive variables at baseline: gender, puberty, waist, HOMA-IR. Significant results are shown in bold.

In a logistic binary regression analysis, the OR for having a follow-up HbA1c value > 5.3% (34 mmol/mol) (i.e., the median value for all the studied subjects) was 0.193 (95% CI 0.089-0.420; p<0.0001) in subjects with baseline BTR above the median for the whole group, adjusted for baseline age and BMI. In ROC curve analysis, BTR was found to have an acceptable discriminatory accuracy for follow-up HbA1c prediction in all subjects (AUC of 0.680) and a good discriminatory accuracy in the higher age group (AUC of 0.756) (**Figure 3B**).

### Age interactions

The interactions of age in the association between HbA1c and both TB and BTR at baseline and at follow-up were also tested. ANCOVA analysis showed age interactions with p values < 0.15 in the association between TB and HbA1c at follow up (p=0.067) and in the association between BTR and HbA1c at baseline (p=0.120).

## Discussion

We show that TB and BTR are independently associated with HbA1c in apparently healthy children, in both cross-sectional and longitudinal analyses. These associations were noteworthy in older children.

In line with our results, several studies in healthy adults have reported negative associations between bilirubin and HbA1c (3, 10, 29). In children, a study in type 1 diabetic subjects showed that bilirubin was also inversely associated with HbA1c (30). Lin et al. showed an inverse association between serum bilirubin and metabolic syndrome in children and adolescents and showed also inverse associations with insulin and HOMA-IR. The authors, however, did not report HbA1c levels (17). In our study, we also observed associations between TB and HOMA-IR and HOMA-B but they did not remain significant in multivariate linear models. Further studies assessing more dynamic measurements of insulin resistance and secretion are warranted.

Total serum bilirubin is composed of direct and indirect forms of bilirubin. Studies on the effects of bilirubin on cardiovascular disease and metabolic syndrome have typically focused on total serum bilirubin (3–5, 8). Even though some studies have also described and inverse relationship between direct bilirubin and metabolic syndrome in adult patients (31, 32) we have not found independent associations assessing either of the bilirubin fractions.

Studies in adult patients showed that type 2 diabetic patients had lower bilirubin values (33) and higher concentrations of serum bilirubin were associated with decreased prevalence of type 2 diabetes (9, 10). Moreover, diabetic patients with Gilbert’s syndrome (who have mild elevations of bilirubin) had better HbA1c values and a more favorable profile of cardiovascular risk markers compared to those without Gilbert’s syndrome (34). Apart from a longitudinal study that described a negative association between serum bilirubin levels and incident type 2 diabetes in a 4-year period in healthy Korean men (11), the majority of published studies to-date are cross-sectional and cannot draw conclusions regarding the temporal nature of the association.

Several possible mechanisms have been suggested for the association of bilirubin with HbA1c values, metabolic syndrome and diabetes, although the underlying exact mechanism is still not known (7). Insulin resistance and impaired glucose tolerance are all accompanied by oxidative stress and subclinical inflammation state (4). Oxidative stress contributes to progressive β-cell damage and plays an important role in type 2 diabetes, and also in type 1 diabetes (35), which is mainly caused by autoimmunity and inflammation. As bilirubin is a potent anti-inflammatory and anti-oxidant agent, capable of protecting cells from a, 10000-fold excess of oxidants (36), it seems reasonable to suggest that the inverse relationship between bilirubin, insulin resistance and diabetes might result from these protective properties. Experimental studies in mice and rats with type 2 diabetes and insulin resistance revealed that the up-regulation of bilirubin synthesis by induction of heme-oxigenase ameliorates glucose metabolism and insulin sensitivity in rodents (37, 38). Another experimental study performed in a type 2 diabetic rat model demonstrated that the administration of biliverdin (a precursor of bilirubin) improved glucose tolerance by attenuating the oxidative stress in β-cells and protecting them from destruction (39). Cohen et al. demonstrated that bilirubin can modulate the expression of glucose transporter-1 and increase the rate of glucose uptake in vascular endothelial cells (40) and Lin et al. postulate that a low human biliverdin, and consequently bilirubin activity, could not only result in unopposed tissue oxidative stress but also cause dysregulation of insulin signaling, which is a hallmark of metabolic syndrome (17). Higher HbA1c itself is significantly associated with increased oxidative stress. Therefore, another explanation could be that mildly elevated serum bilirubin may inhibit the glycation of hemoglobin by reducing oxidative stress (29). Further studies assessing the relationship between HbA1c and markers of oxidative stress are required to elucidate this issue.

Several authors have shown that serum triglycerides are positively associated with insulin resistance and metabolic syndrome (15, 16, 21, 24) and negatively associated with bilirubin (4, 5, 9, 18, 31). Dyslipidemia and insulin resistance are strongly linked but it is largely unknown which of these factors is the precursor, or whether the temporal relationship between them is bidirectional. Several studies in humans support the theory that hypertriglyceridemia and low HDL cholesterol may be causal factors of insulin resistance; epidemiological studies have shown that hypertriglyceridemia can predict the risk of developing type 2 diabetes, intervention studies have suggested that lowering triglyceride treatments could delay the onset of insulin resistance in adult patients with coronary artery disease or attenuate the declining of β-cell function in adult diabetic patients and genetic studies have shown that mutations in genes involved in lipoprotein pathways that have no direct role in insulin resistance can cause dyslipidemia and consequently lead to insulin resistance (16). A cross-lagged path analysis performed by Han et al. demonstrated that changes in triglycerides and HDL cholesterol precede peripheral insulin resistance (15). The underlying mechanisms of the association between triglycerides and HDL cholesterol and insulin resistance are not clear but it seems that the excess of intracellular lipids (free fatty acids, triacylglycerol and triglyceride-rich lipoproteins) in skeletal muscle, liver and heart muscle results in insulin resistance and altered organ function. Cell cultures and rodent models have demonstrated that lipotoxicity, which is associated with hypertriglyceridemia, increases β-cell apoptosis and decreases β-cell insulin secretion (41). However, the effects of hypertriglyceridemia on human β-cell function remain still unclear and more research is needed. In our study bilirubin showed negative associations with TG/HDL ratio at follow-up. The TG to HDL ratio has been associated previously with IR in obese white boys and girls (42). There are also other studies that use TG/HDL ratio as a marker of insulin resistance in minorities (43–45). In reference to insulin sensitivity, experimental data in mice models showed that bilirubin administration improves hyperglycemia and obesity by increasing insulin sensitivity and suggest that bilirubin or bilirubin-increasing drugs might be useful as an insulin sensitizer for the treatment of obesity-induced insulin resistance and type 2 diabetes based on its profound anti-ER stress and anti-inflammatory properties (46). In humans, the relationships between bilirubin and insulin sensitivity varied according to the different types of bilirubin and serum indirect bilirubin has been suggested to be a protective factor that enhances insulin sensitivity (47). In our study, bilirubin and BTR showed negative associations with HOMA-B, but they did not remain significant in multivariate linear models.

Our results showed that the associations between HbA1c and both total bilirubin and the bilirubin-to-triglycerides ratio are influenced by age and are stronger in older children. We speculate that this age-dependent association could be related to a less favorable metabolic profile (higher insulin resistance and TG/HDL ratio) at an older age in our subjects (48).

The major strength of our study is its longitudinal nature, suggesting a causal relationship between bilirubin and the bilirubin-to-triglycerides ratio and HbA1c levels over time. Unfortunately, some children were lost to follow-up, but it may probably not influence our results since there were no differences in baseline clinical or laboratory assessments between lost-to-follow-up children and the rest of the cohort. Another limitation could be that our study did not include an oral glucose tolerance test, which would have been helpful to further characterize the associations with glucose tolerance or insulin secretion. However, HbA1c is clinical parameter that describes the extent of glucose tolerance over a long period and therefore may be a more robust indicator of glycemia than a parameter describing it in a given moment only, such as during an oral glucose tolerance test (49). We also acknowledge that the children in our study were rather overweight at baseline (mean BMI-SDS of 1.03 ± 0.10). This is because the study was focused on cardiovascular risk factors in children. However, all the associations herein described were independent of BMI.

Another limitation of our study is the low r^2^ values obtained in multivariate models, thus meaning that TB and BTR are not the only parameters involved in the prediction of HbA1c levels. The anti-aging gene Sirtuin 1 could be a novel parameter that together with TB and BTR would predict glucose tolerance in healthy children. Indeed, the role of Sirtuin 1 is critical to glucose tolerance and the metabolic syndrome in children and Sirtuin 1 dysregulation is linked to diabetes and various chronic diseases (50–52). Sirtuin 1 controls beta cell insulin production and glucose regulation and is important to triglyceride metabolism and the prevention of NAFLD (53, 54). Bilirubin has been shown to regulate Sirtuin 1 expression with relevance to triglyceride accumulation and NAFLD (55). The role of bilirubin and Sirtuin 1 on the prediction of glucose tolerance in healthy children should be elucidated in future studies.

In summary, to the best or our knowledge, we show for the first time that bilirubin and the bilirubin-to-triglycerides ratio are independently associated with HbA1c and predict its changes over time in healthy children. Our results indicate that bilirubin and the bilirubin-to-triglycerides ratio may be useful parameters in studies of glucose tolerance in healthy children.

## Data availability statement

The data analyzed in this study is subject to the following licenses/restrictions: The datasets generated and analyzed during the current study are available from the corresponding author on reasonable request. Requests to access these datasets should be directed to AL-B, alopezbermejo@idibgi.org and/or JB, jbassols@idibgi.org.

## Ethics statement

The studies involving humans were approved by Institutional Review Board of Dr. Josep Trueta Hospital. The studies were conducted in accordance with the local legislation and institutional requirements. Written informed consent for participation in this study was provided by the participants’ legal guardians/next of kin.

## Author contributions

EP-C: Conceptualization, Data curation, Formal Analysis, Validation, Writing – original draft, Writing – review & editing. SN-C: Formal Analysis, Writing – review & editing. BR-P: Formal Analysis, Writing – review & editing. BM-P: Data curation, Methodology, Writing – review & editing. AG-V: Data curation, Methodology, Writing – review & editing. GC-B: Conceptualization, Data curation, Formal Analysis, Writing – review & editing. MN: Data curation, Writing – review & editing, Methodology. AP-P: Writing – review & editing, Data curation. FZ: Conceptualization, Writing – review & editing. LI: Conceptualization, Writing – review & editing. JB: Conceptualization, Formal Analysis, Funding acquisition, Methodology, Supervision, Writing – original draft, Writing – review & editing. AL-B: Conceptualization, Formal Analysis, Funding acquisition, Methodology, Supervision, Writing – original draft, Writing – review & editing.
