# Supplementary material for: Total bilirubin and bilirubin-to-triglycerides ratio predict changes in glycated hemoglobin in healthy children
Source: Front Endocrinol (Lausanne). 2023 Dec 1;14:1303597. doi: 10.3389/fendo.2023.1303597 (PMC10722262; doi:10.3389/fendo.2023.1303597)
Supplement: Supplementary file 1 [file Table_1.pdf]

Supplemental Table S1: Baseline assessments in the studied subjects as a whole and in subgroups thereof according to age.

	**All subjects (n=246)**	**1st Tertile (<7.8 years) (n=82)**	**2nd Tertile (7.8-9.6 years) (n=82)**	**3rd Tertile (>9.7 years) (n=82)**	**p for trend**
**Clinical assessments**					
Age (yr)	8.8 ± 0.1	6.9 ± 0.1	8.8 ± 0.1	10.8 ± 0.1	**<0.0001**
Gender (%female)	47	51	48	42	0.211
Puberty (%≥T2)	17	0	16	39	**<0.0001**
Weight (Kg)	42 ± 1	29.5 ± 1	40 ± 1	56 ± 2	**<0.0001**
Weight-SDS	1.2 ± 0.1	0.9 ± 0.2	1.1 ± 0.2	1.6 ± 0.2	**0.004**
Height (cm)	137 ± 1	125 ± 1	138 ± 1	149 ± 1	**<0.0001**
Height-SDS	0.76 ± 0.07	0.66 ± 0.13	0.80 ± 0.12	0.81 ± 0.12	0.378
BMI (kg/m^2)	21.3 ± 0.3	18.6 ± 0.5	20.9 ± 0.5	24.5 ± 0.6	**<0.0001**
BMI-SDS	1.03 ± 0.10	0.71 ± 0.18	0.92 ± 0.16	1.47 ± 0.16	**0.001**
Waist (cm)	70 ± 1	61 ± 1	70 ± 1	80 ± 1	**<0.0001**
SBP (mmHg)	109 ± 1	104 ± 1	110 ± 1	113 ± 1	**<0.0001**
DBP (mmHg)	63 ± 1	59 ± 1	63 ± 1	66 ± 1	**<0.0001**
**Laboratory assessments**					
TB (mg/dl)	0.13 (0.08-0.20)	0.14 (0.08-0.21)	0.12 (0.07-0.18)	0.15 (0.09-0.22)	0.458
DB (mg/dl)	0.09 (0.06-0.13)	0.09 (0.06-0.13)	0.09 (0.07-0.13)	0.10 (0.07-0.13)	0.339
IB (mg/dl)	0.05 (0.01-0.09)	0.05 (0.01-0.10)	0.04 (0.01-0.07)	0.05 (0.02-0.10)	0.485
TG (mg/dl)	54 (40-78)	47 (36-66)	56 (41-78)	62 (45-92)	**<0.0001**
BTR (%)	0.20 (0.10-0.40)	0.26 (0.17-0.43)	0.21 (0.09-0.39)	0.22 (0.11-0.43)	0.202
Glucose (mg/dl)	86 (83-91)	84 (82-89)	88 (84-93)	87 (83-92)	**0.014**
Insulin (mIU/L)	5.3 (2.2-9.7)	2.4 (0.5-5.8)	5.6 (2.6-9.3)	8.4 (4.5-12.4)	**<0.0001**
HbA1c (%)	5.3 (5.1-5.5)	5.3 (5.0-5.4)	5.3 (5.1-5.5)	5.4 (5.2-5.5)	**<0.0001**
HbA1c (mmol/mol)	34 (32-37)	34 (31-36)	34 (32-37)	36 (33-37)	**<0.0001**
Hb (g/dL)	12.9 (12.8-12.9)	12.8 (12.6-12.9)	12.9 (12.7-13.0)	12.9 (12.8-13.1)	0.294
HOMA-IR	1.2 (0.4-2.1)	0.5 (0.1-1.3)	1.2 (0.6-2.0)	1.8 (1.0-2.7)	**<0.0001**
HOMA-B	27.9 (24.3-31.5)	16.3 (12.6-19.9)	25.6 (21.3-29.9)	41.8 (33.3-50.3)	**<0.0001**
TC (mg/dL)	162 (144-179)	166 (151-180)	159 (144-170)	161 (136-180)	0.102
LDL (mg/dL)	92 (78-106)	96 (84-108)	90 (74-102)	91 (76-110)	0.575
HDL (mg/dL)	56 (47-65)	57 (50-72)	57 (48-65)	52 (44-60)	**0.001**
TG/HDL	1.2 (1.1-1.4)	0.9 (0.8-1.1)	1.2 (1.0-1.4)	1.6 (1.3-1.8)	**<0.0001**

Results are shown as mean and SEM for Gaussian variables and as median and interquartile range for non-Gaussian variables. T2: Tanner 2; SBP: systolic blood pressure; DBP: diastolic blood

pressure; TB: total bilirubin, TG: triglycerides, DB: direct bilirubin, IB: indirect bilirubin, BTR: bilirubin-to-triglycerides ratio; HbA1c: glycosylated hemoglobin; Hb: hemoglobin; HOMA-IR: homeostasis model assessment insulin resistance; HOMA-B: homeostasis model assessment of beta-cell function; TC: total cholesterol; TG/HDL: triglyceride-to-HDL ratio. Significant results are shown in bold.

Supplemental Table S2: Follow-up assessments in the studied subjects as a whole and in subgroups thereof according to age.

	**All subjects (n=142)**	**Below median <12.9 years (n=71)**	**Above median ≥12.9 years (n=71)**	**p for trend**
**Clinical Assessments**				
Age (yr)	12.9 ± 0.1	11.4 ± 0.1	14.4 ± 0.1	**<0.0001**
Gender (%F)	48	51	45	0.503
Puberty (%≥T2)	70	43	97	**<0.0001**
Weight (Kg)	59 ± 2	47 ± 2	70 ± 2	**<0.0001**
Weight-SDS	0.78 ± 0.12	0.39 ± 0.15	1.2 ± 0.2	**0.001**
Height (cm)	159 ± 1	151 ± 1	166 ± 1	**<0.0001**
Height-SDS	0.45 ± 0.09	0.45 ± 0.14	0.44 ± 0.11	0.940
BMI (kg/m^2)	22.9 ± 0.5	20.5 ± 0.6	25.2 ± 0.7	**<0.0001**
BMI-SDS	0.67 ± 0.13	0.23 ± 0.15	1.12 ± 0.19	**<0.0001**
Waist (cm)	77 ± 1	70 ± 1	83 ± 2	**<0.0001**
SBP (mmHg)	115 ± 1	109 ± 1	121 ± 1	**<0.0001**
DBP (mmHg)	63 ± 1	60 ± 1	65 ± 1	**0.002**
**Laboratory Assessments**				
TG (mg/dl)	57 (44-79)	56 (42-71)	62 (45-82)	0.266
Glucose (mg/dl)	87 (81-91)	87 (81-90)	87 (81-94)	0.412
Insulin (mIU/L)	9.9 (7.0-14.3)	8.9 (6.4-13.5)	11.9 (8.9-15.2)	**0.016**
HbA1c (%)	5.3 (5.1-5.4)	5.3 (5.1-5.4)	5.3 (5.1-5.4)	0.444
HbA1c (mmol/mol)	34 (32-36)	34 (32-36)	34 (32-36)	0.444
Hb (g/dL)	13.5 (13.4-13.7)	13.3 (13.1-13.5)	13.8 (13.5-14.1)	**0.006**
HOMA-IR	2.2 (1.5-3.1)	1.8 (1.4-2.9)	2.5 (1.7-3.2)	**0.015**
HOMA-B	49.1 (44.3-53.9)	45.3 (37.9-52.6)	52.8 (46.5-59.1)	0.120
TC (mg/dl)	152 (134-169)	157 (141-174)	145 (129-162)	**0.008**
LDL (mg/dl)	81 (67-96)	82 (67-92)	78 (64-98)	0.808
HDL (mg/dL)	55 (46-70)	65 (51-78)	49 (41-59)	**<0.0001**
TG/HDL	1.3 (1.1-1.5)	1.2 (0.9-1.4)	1.4 (1.2-1.6)	0.148

Results are shown as mean and SEM for Gaussian variables and as median and interquartile range for non-Gaussian variables. T2: Tanner 2; SBP: systolic blood pressure; DBP: diastolic blood pressure; TG: triglycerides; HbA1c: glycosylated hemoglobin; Hb: hemoglobin; HOMA-IR: homeostasis model assessment insulin resistance; HOMA-B: homeostasis model assessment of beta-cell function; TC: total cholesterol; TG/HDL: triglyceride-to-HDL ratio. Significant results are shown in bold.

Supplemental Table S3: Correlation analyses between baseline total bilirubin and selected variables at baseline and follow-up in the studied subjects.

**Baseline TB**	**All subjects (n=246)**		**1st Tertile <7.8 years (n=82)**		**2nd Tertile 7.8-9.6 years (n=82)**		**3rd Tertile >9.7 years (n=82)**	
**Baseline parameters**	r	p	r	p	r	p	r	p
Age	0.046	0.471	0.064	0.567	-0.209	0.060	0.104	0.354
Weight-SDS	-0.093	0.145	0.005	0.962	-0.189	0.089	-0.164	0.141
Height-SDS	0.046	0.471	0.115	0.302	0.022	0.848	-0.006	0.956
BMI-SDS	**-0.135**	**0.034**	-0.048	0.666	-0.215	0.052	-0.214	0.053
Waist	-0.119	0.063	-0.054	0.631	-0.124	0.269	**-0.312**	**0.004**
SBP	-0.113	0.077	0.038	0.731	**-0.260**	**0.020**	-0.171	0.125
DBP	-0.100	0.118	-0.180	0.106	-0.201	0.074	0.004	0.969
Glucose	-0.091	0.156	-0.191	0.085	-0.038	0.734	-0.013	0.908
Insulin	**-0.172**	**0.007**	**-0.220**	**0.047**	**-0.259**	**0.019**	-0.142	0.204
HOMA-IR	**-0.175**	**0.006**	**-0.230**	**0.038**	**-0.259**	**0.019**	-0.141	0.208
HOMA-B	**-0.168**	**0.008**	-0.209	0.060	**-0.259**	**0.019**	-0.142	0.202
HbA1c	**-0.219**	**0.001**	-0.134	0.232	**-0.252**	**0.023**	**-0.334**	**0.002**
TG	-0.069	0.282	0.075	0.502	-0.170	0.128	-0.140	0.209
HDL	**0.132**	**0.038**	0.075	0.504	0.168	0.132	0.200	0.072
TG/HDL	-0.084	0.189	-0.074	0.509	-0.154	0.167	-0.162	0.147

**Baseline TB**	**All subjects (n=142)**		**Below median <12.9 years (n=71)**		**Above median ≥12.9 years (n=71)**	
**Follow-up parameters**	r	p	r	p	r	p
Age	0.018	0.830	-0.085	0.483	0.089	0.461
Weight-SDS	-0.078	0.355	-0.129	0.285	-0.050	0.677
Height-SDS	0.082	0.335	0.107	0.376	0.047	0.700
BMI-SDS	-0.142	0.091	-0.221	0.064	-0.100	0.404
Waist	-0.113	0.181	-0.205	0.089	-0.081	0.500
SBP	-0.056	0.507	-0.141	0.242	-0.016	0.898
DBP	-0.128	0.131	-0.128	0.287	-0.149	0.217
Glucose	-0.149	0.077	-0.183	0.126	-0.127	0.291
Insulin	**-0.208**	**0.013**	-0.225	0.060	-0.205	0.087
HOMA-IR	**-0.222**	**0.008**	**-0.238**	**0.046**	-0.221	0.064
HOMA-B	**-0.194**	**0.020**	-0.206	0.084	-0.179	0.135
HbA1c	**-0.269**	**0.001**	-0.177	0.139	**-0.377**	**0.001**
TG	**-0.271**	**0.001**	**-0.323**	**0.006**	-0.211	0.077
HDL	0.163	0.052	**0.266**	**0.025**	0.09	0.454
TG/HDL	**-0.257**	**0.002**	**-0.284**	**0.017**	-0.212	0.076

Results are shown according to age categories. P and r values are from Pearson correlation analyses. Significant results are shown in bold.

Supplemental Table S4: Effect sizes per 1-unit increase in baseline total bilirubin (mg/dL) in the studied subjects as a whole and in subgroups thereof according to age at baseline and at follow-up.

**Baseline HbA1c**	**All subjects (n=246)**			**1st Tertile <7.8 years (n=82)**			**2nd Tertile 7.8-9.6 years (n=82)**			**3rd Tertile >9.7 years (n=82)**		
**Baseline parameters**	B	error	p	B	error	p	B	error	p	B	error	p
TB	-0.4	0.1	**0.002**	-	-	-	-0.7	0.2	**0.006**	-0.6	0.2	**0.003**

**Follow-up HbA1c**	**All subjects (n=142)**			**Below median <12.9 years (n=71)**			**Above median ≥12.9 years (n=71)**		
**Baseline parameters**	B	error	p	B	error	p	B	error	p
TB	-0.4	0.2	**0.018**	-	-	-	-0.5	0.2	**0.019**

Unadjusted multivariate regression coefficient (B), SE and p value are shown.

Supplemental Table S5: Correlation analyses between baseline bilirubin-to-triglycerides ratio and selected variables in the studied subjects at baseline and follow-up.

**Baseline BTR**	**All subjects (n=246)**		**1st Tertile <7.8 years (n=82)**		**2nd Tertile 7.8-9.6 years (n=82)**		**3rd Tertile >9.7 years (n=82)**	
**Baseline parameters**	r	p	r	p	r	p	r	p
Age	-0.087	0.173	0.008	0.944	-0.187	0.093	0.043	0.700
Weight SDS	**-0.270**	**<0.0001**	-0.143	0.199	**-0.340**	**0.002**	**-0.324**	**0.003**
Height SDS	-0.027	0.677	0.080	0.474	-0.067	0.550	-0.070	0.529
BMI SDS	**-0.324**	**<0.0001**	-0.211	0.057	**-0.362**	**0.001**	**-0.398**	**<0.0001**
Waist	**-0.332**	**<0.0001**	-0.211	0.057	**-0.298**	**0.007**	**-0.478**	**<0.0001**
SBP	**-0.186**	**0.003**	0.045	0.686	**-0.356**	**0.001**	-0.170	0.126
DBP	**-0.233**	**<0.0001**	**-0.218**	**0.049**	**-0.339**	**0.002**	-0.107	0.337
Glucose	**-0.144**	**0.024**	**-0.257**	**0.020**	-0.137	0.219	0.023	0.837
Insulin	**-0.381**	**<0.0001**	**-0.411**	**<0.0001**	**-0.417**	**<0.0001**	**-0.325**	**0.003**
HOMA-IR	**-0.384**	**<0.0001**	**-0.423**	**<0.0001**	**-0.420**	**<0.0001**	**-0.318**	**0.004**
HOMA-B	**-0.283**	**<0.0001**	**-0.398**	**<0.0001**	**-0.392**	**<0.0001**	**-0.338**	**0.002**
HbA1c	**-0.313**	**<0.0001**	-0.133	0.233	**-0.307**	**0.005**	**-0.452**	**<0.0001**
HDL	**0.365**	**<0.0001**	**0.292**	**0.008**	**0.390**	**<0.0001**	**0.391**	**<0.0001**

**Baseline BTR**	**All subjects (n=142)**		**Below median <12.9 years (n=71)**		**Above median ≥12.9 years (n=71)**	
**Follow-up parameters**	r	p	r	p	r	p
Age	-0.093	0.269	-0.144	0.232	0.052	0.667
Weight SDS	**-0.226**	**0.007**	-0.221	0.064	-0.204	0.089
Height SDS	-0.019	0.823	0.015	0.901	-0.062	0.608
BMI SDS	**-0.276**	**0.001**	**-0.295**	**0.013**	**-0.239**	**0.045**
Waist	**-0.270**	**0.001**	**-0.296**	**0.013**	-0.232	0.052
SBP	**-0.156**	**0.065**	-0.199	0.097	-0.077	0.526
DBP	**-0.255**	**0.002**	-0.204	0.088	**-0.278**	**0.020**
Glucose	**-0.213**	**0.011**	**-0.258**	**0.030**	-0.179	0.136
Insulin	**-0.328**	**<0.0001**	**-0.249**	**0.036**	**-0.356**	**0.002**
HOMA-IR	**-0.348**	**<0.0001**	**-0.306**	**0.009**	**-0.376**	**0.001**
HOMA-B	**-0.327**	**<0.0001**	**-0.291**	**0.015**	**-0.313**	**0.009**
HbA1c	**-0.307**	**<0.0001**	-0.151	0.209	**-0.479**	**<0.0001**
HDL	**0.266**	**0.001**	**0.375**	**0.001**	0.128	0.288

Results are shown according to age categories. p and r values are from Pearson correlation analyses. Significant results are shown in bold.

Supplemental Table S6: Effect sizes per 1-unit increase in baseline bilirubin-to-triglycerides ratio (%) in the studied subjects as a whole and in subgroups thereof according to age at baseline and at follow-up.

**Baseline HbA1c**	**All subjects (n=246)**			**1st Tertile <7.8 years (n=82)**			**2nd Tertile 7.8-9.6 years (n=82)**			**3rd Tertile >9.7 years (n=82)**		
**Baseline parameters**	B	error	p	B	error	p	B	error	p	B	error	p
**BTR**	-0.3	0.1	**<0.001**	-	-	-	-0.3	0.1	**0.014**	-0.4	0.1	**<0.001**

**Follow-up HbA1c**	**All subjects (n=142)**			**Below median <12.9 years (n=71)**			**Above median ≥12.9 years (n=71)**		
**Baseline parameters**	B	error	p	B	error	p	B	error	p
**BTR**	-0.2	0.1	**0.014**	-	-	-	-0.4	0.1	**<0.001**

Unadjusted multivariate regression coefficient (B), SE and p value are shown.
